# Intensified Tuberculosis Case-Finding in HIV-Positive Adults Managed at Ethiopian Health Centers: Diagnostic Yield of Xpert MTB/RIF Compared with Smear Microscopy and Liquid Culture

**DOI:** 10.1371/journal.pone.0085478

**Published:** 2014-01-22

**Authors:** Taye T. Balcha, Erik Sturegård, Niclas Winqvist, Sten Skogmar, Anton Reepalu, Zelalem Habtamu Jemal, Gudeta Tibesso, Thomas Schön, Per Björkman

**Affiliations:** 1 Infectious Disease Research Unit, Department of Clinical Sciences Malmö, Lund University, Malmö, Sweden; 2 Ministry of Health, Addis Ababa, Ethiopia; 3 Clinical Microbiology, Regional and University Laboratories, Region Skåne, Sweden; 4 Regional Department of Infectious Disease Control and Prevention, Malmö, Sweden; 5 Oromia Health Bureau, Addis Ababa, Ethiopia; 6 Columbia University Mailman School of Public Health, International Center for AIDS Care and Treatment Programs- Ethiopia, Addis Ababa, Ethiopia; 7 Department of Medical Microbiology, Faculty of Health Sciences, Linköping University, Sweden; 8 Department of Clinical Microbiology and Infectious diseases, Kalmar County Hospital, Sweden; National Institute of Medical Research, United Kingdom

## Abstract

**Background:**

Detection of active tuberculosis (TB) before antiretroviral therapy (ART) initiation is important, but optimal diagnostic methods for use in resource-limited settings are lacking. We assessed the prevalence of TB, evaluated the diagnostic yield of Xpert MTB/RIF in comparison with smear microscopy and culture, and the impact of Xpert results on clinical management in HIV-positive adults eligible for ART at health centers in a region of Ethiopia.

**Methods:**

Participants were prospectively recruited and followed up at 5 health centers. Trained nurses collected data on socio-demographic characteristics, medical history and symptoms, and performed physical examination. Two paired morning sputum samples were obtained, and lymph node aspirates in case of lymphadenopathy. Diagnostic yield of Xpert MTB/RIF in sputum was compared with smear microscopy and liquid culture.

**Results:**

TB was diagnosed in 145/812 participants (17.9%), with bacteriological confirmation in 137 (16.9%). Among bacteriologically confirmed cases, 31 were smear-positive (22.6%), 96 were Xpert-positive (70.1%), and 123 were culture-positive (89.8%). Xpert MTB/RIF increased the TB detection rate by 64 cases (47.4%) compared with smear microscopy. The overall sensitivity of Xpert MTB/RIF was 66.4%, and was not significantly lower when testing one compared with two samples. While Xpert MTB/RIF was 46.7% sensitive among patients with CD4 cell counts >200 cells/mm^3^, this increased to 82.9% in those with CD4 cell counts ≤100 cells/mm^3^. Compared with Xpert-positive TB patients, Xpert-negative cases had less advanced HIV and TB disease characteristics.

**Conclusions:**

Previously undiagnosed TB is common among HIV-positive individuals managed in Ethiopian health centers. Xpert MTB/RIF increased TB case detection, especially in patients with advanced immunosuppression. An algorithm based on the use of a single morning sputum sample for individuals with negative sputum smear microscopy could be considered for intensified case finding in patients eligible for ART. However, technical and cost-effectiveness issues relevant for low-income countries warrant further study.

## Introduction

The recent achievements in mortality reduction in HIV-positive individuals through the scale up of antiretroviral therapy (ART) in sub-Saharan Africa have been dented by a high occurrence of TB, which remains the leading cause of death [1], [2]. Studies from South Africa, Uganda and Tanzania have shown unrecognized active TB in 7–31% of patients starting ART [3]–[5]. Atypical disease manifestations and lack of suitable diagnostic tools contribute to suboptimal TB case finding [5]–[7]. Delayed diagnosis of TB increases the risk both of mortality and of immune reconstitution inflammatory syndrome [4], [8] in patients receiving ART, and is also associated with TB transmission in health facilities [9] and in the community [10]. In addition, isoniazid preventive therapy (IPT) may be inadvertently administered to patients with active TB [11].

The World Health Organization (WHO) recommends regular symptom screening for TB for HIV-positive individuals in TB-endemic settings [12]. Although the overall sensitivity of the current algorithm is 79%, its specificity is estimated at 50% [13], indicating that a majority of patients identified will need further investigations to ascertain their TB status. In most settings, especially at primary health care level, the century-old acid fast bacilli (AFB) smear microscopy is currently the only available method to diagnose TB. In HIV-positive individuals the performance of sputum smear microscopy is unsatisfactory; the sensitivity of this technique in HIV-positive individuals starting ART in South Africa was 22% and 26% using one and two samples, respectively [14].

Mycobacterial culture is generally considered to have the highest sensitivity for detection of *Mycobacterium tuberculosis* (Mtb), and is generally used for reference in evaluation of alternative techniques [15]; it is also the only method that can give information on antibiotic sensitivity patterns. However, widespread use of culture is compromised by protracted result delivery [14], high upfront cost and strict requirement of advanced biosafety precautions [6], [16]. Furthermore, culture results may be affected by antibiotic therapy, as well as storage and transport conditions [17]. In resource-limited settings, mycobacterial culture therefore mainly has a role for surveillance and for cases with suspected drug resistance.

In December 2010, the WHO approved a new molecular assay-based test, Xpert MTB/RIF, for the diagnosis of TB in HIV-positive individuals [18]. This assay is an all inclusive molecular test that uses real-time polymerase chain reaction (PCR) to amplify a Mtb-specific sequence of the RNA polymerase gene (*rpoB*) [19]. This gene is probed with molecular beacons to detect rifampicin resistance mutations which can be used as surrogate markers for multidrug-resistant TB (MDR-TB) [19]. A 2013 Cochrane review by Steingart et al summarized several evaluations of Xpert MTB/RIF, demonstrating high overall sensitivity and specificity, albeit mostly conducted in individuals with suspected pulmonary TB [20]. Good diagnostic performance has also been shown for TB lymphadenitis [21].

The lower detection limit of the assay has been estimated at 131 CFU/mL [19]; hence. the sensitivity of Xpert MTB/RIF is directly related to the bacterial burden in the specimen, and consequently correlates with sputum smear status [22]. The higher occurrence of paucibacillary forms of pulmonary TB in HIV-positive individuals could explain the lower sensitivity found in such patients [22], [23]. Yet, the lack of accurate diagnostic methods and the poor prognosis of TB in HIV-infected subjects suggest that these are the ones who may benefit most from the introduction of Xpert MTB/RIF. This test has been evaluated for TB screening before starting ART in a cohort from South Africa; Xpert MTB/RIF increased detection rates of TB by 45% compared with smear microscopy [14]. Although sensitivity was reduced for smear-negative TB, patients detected by Xpert MTB/RIF were characterized by more advanced immunosuppression as compared to Xpert-negative cases [24].

Despite promising preliminary results and the WHO endorsement, the actual role of Xpert MTB/RIF in resource-constrained settings endemic for HIV and TB is unclear, especially considering drawbacks of this method such as cost, requirement of ambient temperature below 28°C, uninterrupted power supply and consumables [25]. In particular, the use of this test for TB diagnosis in HIV-positive individuals managed at health centers – where around 60% of TB patients globally are treated [26] - has not been evaluated.

In 2011 Ethiopia had the 7^th^ highest level of incident TB among the 22 high burden countries worldwide [27]. The reported rate of HIV infection among TB patients was 18% in 2008 [28]. Data on the prevalence of bacteriologically confirmed TB among HIV-positive individuals in Ethiopia is limited to a hospital-based study conducted in the capital Addis Ababa in 2006, in which 32 of 438 study participants (7%) were found to have positive sputum cultures for TB [29].

Access to ART has increased rapidly in Ethiopia since 2006, when decentralization of HIV care to health centers was introduced [30]. The proportion of patients receiving ART through health centers has increased in Ethiopia, and is expected to rise further [31].

In this study, we have determined the prevalence of TB among HIV-positive adults eligible to start ART at public health centers in an uptake area in central Ethiopia. We have also evaluated the diagnostic yield of Xpert MTB/RIF compared with sputum smear microscopy, using liquid culture for reference, as well as the impact of Xpert MTB/RIF testing on patient management.

## Materials and Methods

### Settings

This study was conducted in all public health centers (Mojo, Adama, Geda, Dhera and Wolenchiti) providing ART services for residents in Adama town and adjacent rural and suburban districts of Oromia Region of Ethiopia (total population of the uptake area around 600,000). These 5 health centers are located on the highway connecting Addis Ababa and Djibouti, which is considered to be a high risk corridor for HIV infection in Ethiopia. In 2005, Adama, the major town on the highway, had a HIV prevalence of 9% as compared to the national prevalence of 3.5% [32].

In health centers, nurses or health officers (with 4 years of academic training) are entirely in charge of patient care, including ART initiation and follow up. The National Program Guidelines in practice at the time of this study recommended ART initiation in all patients with CD4 cell counts of 200 cells/mm^3^ or lower; as well as for individuals in WHO clinical stage 3 with CD4 cell counts of 350 cells/mm^3^ or lower, and in patients in WHO clinical stage 4 regardless of CD4 cell level [33].

### Study Participants

Individuals aged 18 years or greater were eligible for inclusion if they were diagnosed with HIV (based on rapid tests), had a previously documented CD4 cell count lower than 350 cells/mm^3^ on at least one occasion, or had WHO stage 4 disease, and resided in the catchment area of any one of the 5 health centers. For inclusion participants were also required to submit at least one morning sputum sample. Patients who were taking ART, or reported previous use of ART, as well as patients who had received TB treatment for more than 2 weeks prior to inclusion were excluded. The presence of symptoms suggestive of TB was neither used as a criterion for inclusion nor exclusion.

### Methods

Consecutive HIV-positive individuals presenting to the study health centers during the inclusion period (October 3, 2011 to March 1, 2013) were screened for eligibility. All study procedures, were performed by health center staff who had received detailed and repeated training on the study protocol.

Following inclusion, the study staff at the respective health center collected information using a standardized paper questionnaire on socio-demographic characteristics, medical history and a range of symptoms potentially associated with TB. Current or previous receipt of IPT and cotrimoxazole prophylaxis was also registered. In addition, structured physical examination was performed. Participants were provided with four separate tubes and asked to submit two paired simultaneously and spontaneously expectorated morning sputa to the health center laboratory on consecutive days. Fine needle aspirates (FNA) were obtained from patients found to have peripheral lymphadenopathy of >1 cm (>2 cm for inguinal lymph nodes) size. In addition, blood samples were obtained for CD4 cell testing and hematological parameters.

TB bacteriological test results were communicated to the respective health center staff, who contacted patients for initiation of TB therapy. The study participants could also be initiated on anti-TB treatment by their health provider in case of strong clinical suspicion and positive radiologic findings in accordance with National TB Program Guidelines [34]. With the exception of the intensified TB case finding and regular monitoring by the research team, all other components of patient care were at the discretion of the attending clinicians. At two months after TB diagnosis, study participants were assessed for initiation of treatment, retention in care and mortality.

Data quality was ensured through weekly monitoring at each site by the investigators (TTB and PB). Data was entered into excel files in real time and cross-checked with hard copies by trained project data clerks.

### TB Diagnosis

Trained laboratory technicians at each health center instructed study participants on how to deliver adequate sputum samples. A volume of 1.5 ml of morning sputum that was free of solid particles was considered to be adequate in volume and quality. In case of inadequate samples, participants were asked to repeat submission. On both mornings, one arbitrarily selected sputum sample was delivered to the Adama Regional Laboratory, where Xpert MTB/RIF assay (Cepheid, Sunnyvale, CA) and smear microscopy were performed. The second sputum sample was sent to International Clinical Laboratories (ICL), a private laboratory located in Addis Ababa, for detection of Mtb using liquid culture. Lymph node samples were obtained using fine needle aspiration by a pathologist at a private laboratory in Adama, and were subjected to culture and Xpert MTB/RIF. Health center laboratory personnel transported sputum samples by car to Adama Regional Laboratory and ICL sample collection site in Adama twice a week with strict adherence to cold chain system (distance of 30 minutes). Participants were instructed to submit sputum samples on these week days in order to avoid storage before testing. From the collection site, samples were transferred to ICL in Addis Ababa on a daily basis (travelling distance of about one and half hours by car).

Adama Regional Laboratory is a regional reference laboratory, and is responsible for external quality assurance for hospitals and health centers in Oromia region. ICL is accredited by the Joint Commission.

Before initiation of the study, hands-on training regarding the operation of the assay was provided to the laboratory technologists performing the Xpert MTB/RIF assay. N-acetyl-L-cysteine and sodium hydroxide (NALC-NaOH) decontaminated sputum and/or lymph node aspirate samples were tested by four-module device Xpert MTB/RIF assay following the manufacturer's instructions. After the manufacturer's reagent was mixed with 1 ml sputum at 2∶1 ratio, 2 ml of the homogenized mixture was transferred into the disposable cartridge and inserted into the GeneXpert instrument. As soon as the test results were ready, they were delivered to the attending clinicians to inform on subsequent patient management. The Xpert MTB/RIF results may be inconclusive due to failure of the internal PCR control (termed “invalid” result), or due to premature interruption of the reaction (termed “error” result); for this study both of these test outcomes were considered as failed tests. Study participants with two failed Xpert results were managed based on the liquid culture results. Uninterrupted power supply was maintained during test performance. Both the instrument and the cartridges were kept at ambient temperature not exceeding 28°C. The instrument was calibrated according to the manufacturer's instructions after it had been in use for a year.

Direct smear microscopy for AFB was performed using Ziehl-Neelsen staining on the same sputum sample that was used for Xpert MTB/RIF. The test results were reported in accordance with WHO/International Union Against Tuberculosis and Lung Diseases recommendations [35]. Smear microscopy was performed by two laboratory technologists (with more than 5 years' experience) throughout the study period. As an internal quality control, blinded cross-checking of slides included in this study was practiced on a weekly basis by the laboratory technologists. When new batches of reagents were prepared for smear microscopy, positive and negative controls were tested before study samples to ensure the quality of the reagent. Daily maintenance of the microscope was conducted.

For external quality control 5 AFB stained and 5 non-stained slides received from the national reference laboratory, EHNRI (Ethiopian Health and Nutrition Research Institute, Addis Abeba) were assessed two times per year. The test panel showed adequate staining procedures and evaluation of prestained slides and the error rate was zero throughout the study period.

BACTEC MGIT (Mycobacterial Growth Indicator Tubes) 960 system (BD Diagnostics, Franklin Lakes, NJ) was used for primary culture of Mtb. The identity of any growth was confirmed by the Capilia TB speciation assay (TAUNS, Numazu, Japan). MGITs were inoculated and incubated for up to 42 days and the culture time-to-positivity (TTP) was recorded. Negative culture results were reported if no growth was detected in MGIT or if growth of a mycobacterium other than MTB was observed. The internal quality control for mycobacterial culture included two negative controls consisting of distilled water in addition to each batch of patient samples. One of the negative controls was used as a quality check for the reagent, and the other negative control was used to monitor the prescence of cross contamination. In addition, the *M. tuberculosis* H37Rv (ATCC 27294) strain was processed as a positive control together with the patient samples once a week. Positive cultures were checked against adjacent culture results to detect any risk of cross contamination. All culture results were logged consecutively.

The laboratory staff at both laboratories was blinded to patient clinical characteristics and the TB test results from the other laboratory.

Complete blood count was done using a Sysmex KX-21 (Sysmex Corporation, Kobe, Japan). Likewise, measurements of CD4 cells counting tests were performed with the use of flow cytometry (FACS Calibur, Becton Dickinson).

### Ethical considerations

Ethical approval was obtained from the National Research Ethics Review Committee at the Ministry of Science and Technology of Ethiopia and the Regional Ethical Review Board, Lund University, Lund, Sweden. All study participants provided written informed consent. An impartial witness confirmed consent received from illiterate study participants.

### Statistical analysis

Participants with at least one positive result by smear microscopy, Xpert MTB/RIF or culture were considered as bacteriologically confirmed TB cases [36]. Participants with clinically suspected TB (according to National TB Program Guidelines), and who initiated TB treatment, were categorized as clinically diagnosed cases. Participants in whom TB was not detected using smear microscopy, Xpert MTB/RIF or liquid culture, and in whom TB was not clinically suspected nor treated empirically were defined as non-TB cases.

For evaluation of the diagnostic performance of Xpert MTB/RIF, comparison was made for smear microscopy, using culture results for reference. This analysis included only participants with at least one sample that had been tested with all three methods. Only results from sputum samples were used for this comparison. Hence, patients with positive TB test results obtained exclusively from lymph node aspirates were excluded, as well as those with clinically diagnosed TB. Since culture was used as a reference for this analysis, patients with contaminated cultures were also excluded; however, we included patients with failed Xpert MTB/RIF results in order to get an accurate estimate of the real-life performance of this method in the study setting.

Statistical analysis was performed using IBM SPSS 20.0 for Windows. Pearson's Chi-Square test was used in univariate analysis in two-by-two tables for dichotomized variables. Comparisons of medians using non-parametric Mann-Whitney's U-test were performed for scale variables that were not visually normally distributed (confirmed by skewness calculation)

Smear microscopy and Xpert MTB/RIF results were compared using liquid culture for reference. Sensitivity, specificity, positive predictive value (PPV), and negative predictive value (NPV) were calculated with 95% confidence intervals (95% CIs). All statistical tests were two-sided with a p-value<0.05 considered significant.

## Results

### Patient characteristics

A total of 886 eligible HIV-positive individuals were initially included. However, for 13 of these (with WHO clinical stage lower than 4) no previous CD4 cell count result below 350 cells/mm^3^ could be identified; hence, these subjects were excluded. Furthermore, an additional 61 participants were excluded since they did not deliver sputum samples.

**Figure 1:**
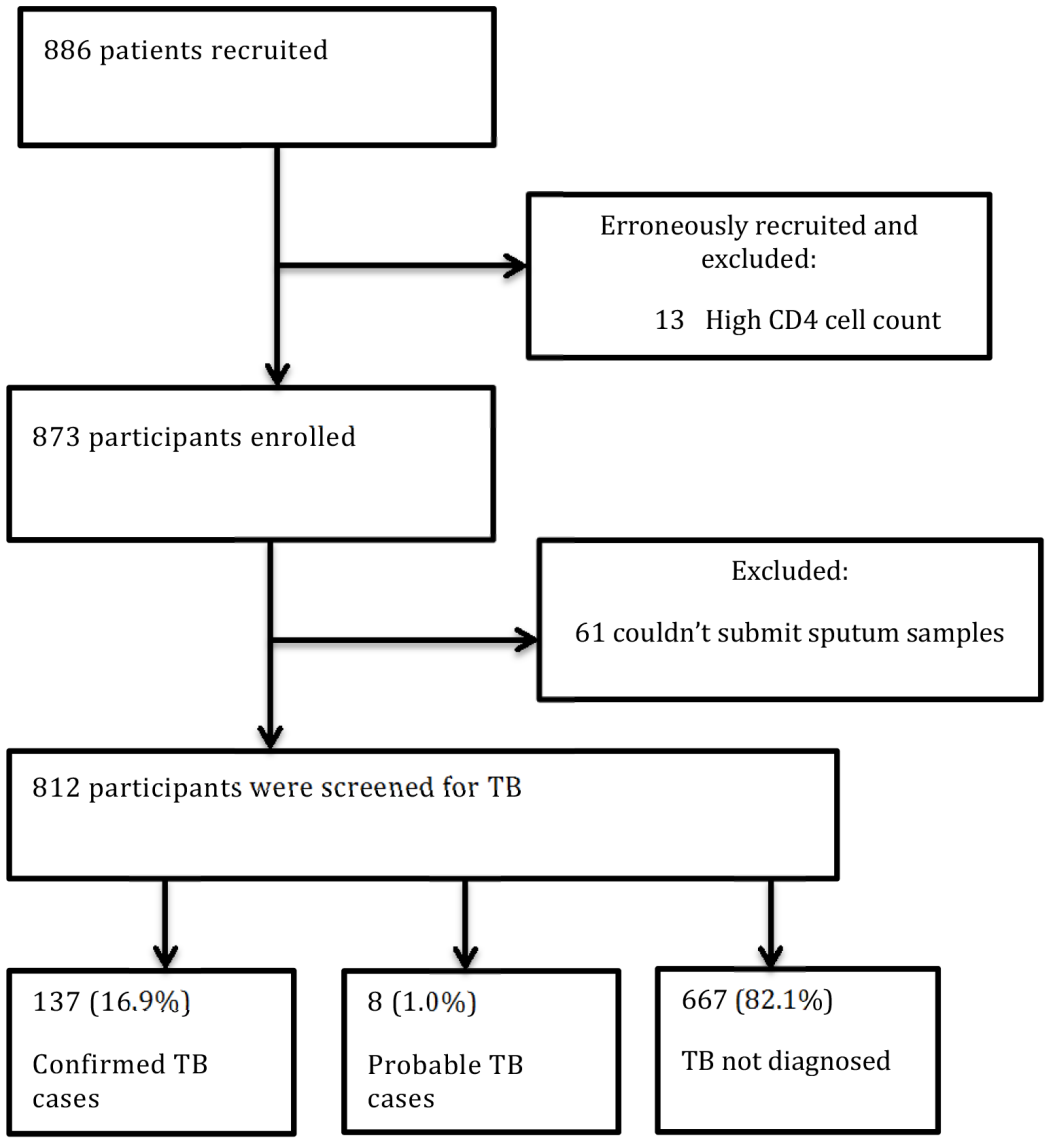
Flow chart of the study participants.

Thus, a total of 812 (336 male, 476 female) study participants with a median age of 32 years (IQR, 28–40) submitted at least one pair of simultaneously expectorated morning sputum samples and were included in the analysis (Figure 1).

The characteristics of the study participants are presented in Table 1. Among them 559 had been enrolled in HIV care before the study was initiated, whereas 253 were newly registered patients at the respective health center. The median CD4 cell count at baseline was 209 cells/mm^3^ (IQR, 118–321). Fifty-one (6.3%) reported a prior episode of TB. Although more common among TB patients (93% vs. 78%; p<0.001), 80% of study participants had a positive WHO symptoms screen (presence of either cough, fever, night-sweating or weight loss).

**Table 1:** Characteristics of the study participants by TB diagnosis.

Characteristics**	Variables	Total No (%)	TB diagnosed* No (%)	TB not diagnosed No (%)	*p*-value
**Total Cohort (%)**		812(100)	145 (17.9)	667 (82.1)	
**Patient Characteristics**					
**Gender**	Male	336 (41)	76 (52)	260 (39)	0.003
	Female	476 (59)	69 (48)	407 (61)	
**Age (years)**	Median age (IQR)	812 (100)	35 (28–42)	32 (28–38)	0.027
**Residence**	Urban	633 (79)	110 (78)	523 (79)	0.678
	Rural	168 (21)	31 (22)	137 (21)	
**BMI (kg/m^2^)**	Median BMI (IQR)	805 (99)	18 (16–20)	19 (18–21)	<0.001
**WHO symptom screening** ***	Positive	646 (80)	134 (93)	512 (78)	<0.001
	Negative	159 (20)	10 (7)	149 (22)	
**WHO clinical stage (prior to TB screening)**	I	141 (17)	12 (8)	129 (19)	<0.001
	II	246 (31)	28 (19)	218 (33)	
	III	322 (40)	71 (49)	251 (38)	
	IV	100 (12)	34 (24)	66 (10)	
**CD4 cell count (cells/mm^3^)**	Median absolute CD4 cell count (IQR)	806 (99)	172 (93–274)	220 (123–328)	<0.001
	CD4 percentage	798 (99)	12 (8–16)	13 (8–17)	0.362
**CD4 cell strata(cells/mm^3^)**					
	>200	426 (53)	57 (39)	369 (55.8)	<0.001
	101–200	223 (28)	47 (33)	176 (26.6)	
	≤100	157 (19)	41 (28)	116 (17.5)	
**Hemoglobin (g/dl)**		761 (94)	10 (9–12)	12 (10–13)	<0.001
**HIV care**					
Enrolment History					
	Newly enrolled	253 (31)	59 (41)	194 (29)	0.006
	In HIV care	559 (69)	86 (59)	473 (71)	
On IPT†					
	Yes	19 (2)	1 (1)	18 (3)	0.145
	No	790 (98)	144 (99)	646 (97)	
On CPT‡					
	Yes	612 (76)	107 (74)	505 (76)	0.552
	No	196 (24)	37 (26)	159 (24)	
**Previous History of TB**	Yes	51 (6)	5 (3)	46 (7)	0.176
	No	749 (94)	139 (97)	610 (93)	

* Including both bacteriologically confirmed and clinical cases of TB.

** Number of missing observations: residence = 11, BMI = 7, WHO Symptom Screening = 7, WHO Clinical Stage = 3, median CD4 cell count = 6, CD4 percentage = 14, CD4 cell strata = 6, Hemoglobin = 51, IPT = 3, CPT = 4, previous TB history = 12.

*** A positive WHO symptom screen is defined as the presence of either cough, fever, night-sweating or weight loss.

† IPT = INH preventive therapy.

‡ CPT = cotrimoxazole preventive treatment.

A total of 1,514 sputum samples were obtained from the study participants. While two paired morning sputum samples were obtained from 702 study participants, 110 participants submitted only one pair of morning sputum samples. Lymph node aspirates were obtained from 22 study participants.

### Tuberculosis Prevalence

TB was diagnosed in 145/812 participants (17.9%); in 137 (16.9%) bacteriological confirmation was obtained. Thirteen (9.5%) of the confirmed TB cases were on TB treatment (for a maximum of 2 weeks) at inclusion. Further, 8 study participants had clinically diagnosed TB (without bacteriological confirmation). TB was not diagnosed in the remaining 667 study participants.

### Bacteriological TB Diagnosis

A total of 1,536 sputum and FNA samples were tested using smear microscopy, Xpert MTB/RIF and liquid culture. Ninety-five (6.2%) of the samples analysed using Xpert MTB/RIF were failed tests (81 error and 14 invalid results), whereas 19 (1.2%) culture samples were contaminated.

Of the 137 bacteriologically confirmed TB patients, 123 (89.8%) were culture positive and 13 (10.2%) were culture-negative. A total of 96 participants were Xpert-positive for TB; among these, 2 were only Xpert-positive in lymph node aspirates, whereas the remaining 94 cases were detected in sputum samples. Rifampicin resistance mutations were detected by Xpert MTB/RIF in 2 cases. Thirty-one patients were sputum smear-positive (Table 2).

**Table 2:** Classification of bacteriologically confirmed TB cases (based on sputum results).

Sputum smear status	Total	Total culture positive	Total Xpert-positive	Xpert-positive culture-positive	Xpert-negative culture-positive	Xpert-positive culture-negative	Xpert-negative culture-negative
Smear-positive n (%)	31 (100)	28 (23)	30 (97)	27 (87)	1 (3)	3 (10)	0 (0)
Smear-negative n (%)	104 (100)	94 (77)	64 (62)	54 (52)	40 (38)	10 (10)	0 (0)
Total n (%)	135 (100)*	122 (90)	94 (70)	81 (60)	41 (30)	13 (10)	0 (0)
Increase in case detection compared to smear microscopy (%)	-	94/135 = 70.1%	64/135 = 47.4%	-	-	-	-

* Patients with clinically diagnosed TB (without bacteriological confirmation) and patients with positive results on lymph node aspirates (but without positive results in sputum) excluded.

Culture results were not available for one Xpert-positive TB patient due to contamination of both morning sputum samples. For one patient both Xpert tests failed; both TB cultures for this subject were negative. Of the 13 Xpert-positive culture-negative TB cases, 3 had received TB treatment for less than 2 weeks when sputum samples were obtained.

The median culture TTP for Xpert-positive and Xpert-negative cases were 10 days (IQR, 7–13) and 13 days (IQR, 8–15), respectively (*p* = 0.041). Based on indications given by the Xpert assay, the following proportions of Xpert-positive patients were observed with regard to assay load: 37 (38.5%) had very low Xpert assay load, 37 (38.5%) had low Xpert assay load, 19 (19.8%) had medium Xpert assay load and 3 (3.1%) had high Xpert assay load. Higher Xpert assay load was associated with AFB smear positivity (*p*<0.001) and shorter median culture TTP (*p*<0.001).

### Diagnostic yield of Xpert MTB/RIF in Sputum Compared to Smear Microscopy and Culture

Using smear microscopy for comparison, Xpert MTB/RIF increased the TB case detection rate by 64 (47.4%) cases. Furthermore, the Xpert MTB/RIF detected 14 (10.2%) TB cases with negative (13 patients) or contaminated culture result (1 patient). Compared to 31 cases detected by sputum smear microscopy (22.6%), 94 cases of pulmonary TB were detected by culture, increasing the case detection rate by 70.1%.

Among 94 participants with Xpert-positive sputum samples 30 (32%) were smear-positive and 64 (68%) were smear-negative. For the 122 culture-positive cases, 28 (23%) and 94 (77%) were smear-positive and negative, respectively. Of 104 smear-negative TB cases, 54 (52%) were positive using both Xpert and culture and 40 (38%) were positive only using culture. All smear-positive TB patients were either Xpert- or culture-positive (Table 2).

Overall, 81 [66.4% (95% CI, 57.2–74.6)] of the culture-positive TB cases were Xpert-positive. The sensitivity using the first morning sputum sample was 57.4% as compared to 67.6% for two samples. In this cohort, the difference in sensitivity between using one or two morning sputum samples was not significant (*p* = 0.729). The sensitivity of Xpert MTB/RIF assay was 96.4% (95% CI, 79.7–99.9) and 57.4% (95% CI, 46.8–67.5) among sputum smear positive and negative TB cases, respectively. Whereas the sensitivity of the Xpert MTB/RIF was 46.7% (95% CI, 31.9–62.0) for participants with CD4 cell counts >200 cells/mm^3^, it increased to 82.9% (95% CI, 65.7–92.9) in patients with CD4 cell counts ≤100 cells/mm^3^. A similar increase in sensitivity was observed with increasing WHO clinical stages (Table 3).

**Table 3:** Comparison of diagnostic yield of direct smear microscopy and Xpert MTB/RIF using culture as reference standard.

Clinical and laboratory characteristics	Variables	Smear microscopy				Xpert MTB/RIF			
		Sensitivity (95% CI)	Specificity (95% CI)	PPV (95% CI)	NPV (95% CI)	Sensitivity (95% CI)	Specificity (95% CI)	PPV (95% CI)	NPV (95% CI)
**Samples**	All samples*	22.9	99.7	93.3	87.8	66.4	98.4	88.0	94.2
		(16.0–31.7)	(98.8–99.9)	(76.4–98.8)	(85.2–90.0)	(57.2–74.6)	(97.0–99.2)	(79.2–93.6)	(92.1–95.8)
	First sample*	19.7	99.7	92.3	87.3	57.4	98.9	90.4	926
		(13.2–28.1)	(98.9–100)	(73.4–98.7)	(84.7–89.6)	(47.8–66.5)	(97.5–99.6)	(80.6–95.8)	(90.2–94.5)
**WHO clinical stage (prior to TB screening)**	I	8.3	100	100	92.1	33.3	100	100	94.1
		(0.4–40.3)	(96.3–100)	(5.4–100)	(85.9–95.8)	(11.2–64.6)	(96.3–100)	(39.5–100)	(88.3–97.3)
	II	22.2	99.5	85.7	91.1	44.4	99.5	92.3	93.5
		(9.3–42.8)	(97.0–100)	(42.0–99.3)	(86.5–94.3)	(26.0–64.4)	(97.0–100)	(62.0–99.6)	(89.3–96.2)
	III	27.1	99.6	94.1	85.7	76.3	97.3	86.5	94.7
		(16.7–40.5)	(97.5–100)	(69.2–99.7)	(81.1–89.4)	(63.1–86.0)	(94.2–98.9)	(73.5–94.0)	(91.1–97.0)
	IV	20.8	100	100	78.7	83.3	95.7	87.0	94.3
		(7.9–42.8)	(93.5–100)	(46.2–100)	(68.4–86.4)	(61.8–94.6)	(86.9–98.9)	(65.3–96.6)	(85.2–98.2)
**CD4 cell count (cells/mm^3^)**	>200	13.3	100	100	90.6	46.7	98.4	77.8	93.9
		(5.5–27.5)	(98.7–100)	(51.6–100)	(87.2–93.2)	(31.9–62.0)	(96.3–99.4)	(57.2–90.7)	(90.9–96.0)
	101–200	23.8	99.4	90.9	84.5	73.8	98.9	93.9	94.1
		(12.5–39.9)	(96.3–100)	(57.1–99.6)	(78.7–89.1)	(57.6–85.7)	(95.5–99.9)	(78.3–99.0)	(89.3–96.9)
	≤100	34.3	99.2	92.3	83.8	82.9	97.5	90.6	95.1
		(19.6–52.3)	(94.7–100)	(62.0–99.6)	(76.4–89.3)	(65.7–92.9)	(92.3–99.4)	(73.8–97.6)	(89.2–98.1)

Patients with clinically diagnosed TB (without bacteriological confirmation) and patients with positive results on lymph node aspirates (but without positive results in sputum) excluded.

Contaminated culture samples were excluded.

* A total of 702 study participants submitted two sputum samples and 110 submitted only one sample.

Using culture for reference, the Xpert MTB/RIF had a specificity of 98.4% (95% CI, 97.0–99.2), and a PPV and a NPV of 88.0% (95% CI, 79.2–93.6) and 94.2% (95% CI, 92.1–95.8), respectively (Table 3).

The sensitivity of smear microscopy was 23% (95% CI, 16.0–31.7) (Table 3). The sensitivity was slightly lower (19.7%;95% CI, 13.2–28.1) when only the first morning sputum sample was used for analysis.

### Characteristics of TB patients with regard to sputum Xpert results and smear status

In this cohort, smear-positive and smear-negative Xpert-positive TB patients had similar clinical characteristics; as shown in table 4, these two groups of patient did not differ significantly with regard to age, gender, BMI, absolute CD4 cell count and WHO clinical stage. In contrast, Xpert-positive and Xpert-negative TB patients displayed significant differences. Xpert-positive TB patients had significantly lower BMI (*p* = 0.043) and absolute CD4 cell count (*p* = 0.006), and higher WHO clinical stage distribution prior to TB screening (*p* = 0.001) (Table 5).

**Table 4:** Characteristics of smear-negative Xpert-positive patients compared to smear-positive cases.

Characteristics	Variables	Total (N = 94)	Smear-positive Xpert-positive (n = 30)	Smear-negative Xpert-positive (n = 64)	*p*-value
**Gender**	Male (%)	54 (57)	16 (53)	38 (59)	0.581
	Female (%)	40 (43)	14 (47)	26 (41)	
**Age**	Median age (IQR)	35 (25–40)	35 (29–40)	36 (28–43)	0.638
**BMI**	Median BMI (IQR)	17.5 (16–19)	17 (15–19)	18 (16–19)	0.259
**CD4 cell count**	Median CD4 cell count (IQR)	94 (100)	128 (63–186)	170 (82–240)	0.179
**CD4 cell strata**	>200 cells/mm^3^ (%)	28 (30)	6 (20)	22 (35)	0.317
	101–200 cells/mm^3^ (%)	33 (35)	11 (37)	22 (34)	
	≤100 cells/mm^3^(%)	33 (35)	13 (43)	20 (31)	
**WHO clinical stage (prior to TB screening)**	I	4 (4)	1 (3)	3 (5)	0.312
	II	13 (14)	7(23)	6 (9)	
	III	53 (56)	16 (54)	37 (58)	
	IV	24 (26)	6 (20)	18 (28)	
**Previous history of TB**	Yes (%)	4 (4)	1 (3)	3 (5)	1.0
	No (%)	89 (96)	29 (97)	60 (95)	

**Table 5:** Characteristics of Xpert-positive and Xpert-negative TB cases (all smear-negative and culture-positive).

Characteristics	Variables	Total (N = 93)	Xpert-positive (n = 54)	Xpert-negative (n = 39)	*p*-value
**Gender**	Male (%)	46 (49)	31 (57)	15 (39)	0.071
	Female (%)	47 (51)	23 (43)	24 (61)	
**Age**	Median age (IQR)	35 (28–43)	36 (28–44)	32 (28–40)	0.261
**BMI**	Median BMI (IQR)	18 (16.5–20.5)	18 (16–19)	20 (16–22)	0.043
**CD4 cell count**	Median CD4 cell count (IQR)	94 (100)	167 (85–212)	211 (145–330)	0.006
**CD4 cell strata**	>200 cells/mm^3^ (%)	38 (41)	16 (30)	22 (56)	0.029
	101–200 cells/mm^3^ (%)	32 (34)	21 (39)	11 (28)	
	≤100 cells/mm^3^ (%)	23 (25)	17 (31)	6 (16)	
**WHO clinical stage (prior to TB screening)**	I	11 (12)	3 (6)	8 (21)	0.001
	II	20 (22)	6 (11)	14 (36)	
	III	43 (46)	30 (56)	13 (33)	
	IV	19 (20)	15 (28)	4 (10)	
**Previous history of TB**	Yes (%)	2 (2)	2 (4)	0 (0)	0.220
	No (%)	90 (98)	51 (96)	39 (100)	

### Patient Management Implications of Xpert MTB/RIF TB Diagnosis

The turnaround time for Xpert MTB/RIF was about 2 hours. In the Xpert-positive TB patients, the median time-to-TB treatment (time from inclusion in the study to initiation of TB treatment) was 9 days (IQR, 6–21) and only 8 (10%) of these initiated ART before starting TB treatment.

The median time-to-TB treatment was 46 days (IQR, 34–60) for Xpert-negative culture-positive TB patients. Consequently, a substantial proportion of Xpert-negative TB cases (49%) started ART before TB treatment (*P*<0.001) (Table 6).

**Table 6:** Short-term implications of Xpert MTB/RIF on patient management; Xpert-positive compared to Xpert-negative TB cases.

Diagnostic test and patient outcome factors		Total	Culture-positive Xpert-Positive (n = 81)	Culture- positive Xpert-negative (n = 40)	*p*-value
**Median time-to-TB treatment (IQR)** *	Days	15 (6–39)	9 (6–21)	46 (34–60)	<0.001
**ART initiated before TB treatment no(%)**	Yes	24 (22)	8 (10)	16 (49)	<0.001
	No	86 (78)	69 (90)	17 (51)	
**Started TB treatment within 2 months of inclusion no(%)**	Yes	96 (78)	73 (90)	23 (55)	<0.001
	No	27 (22)	8 (10)	19 (45)	
**Died within the first 2 months no(%)**	Yes	8 (8)	5 (7)	3 (9)	0.699
	No	96 (92)	67 (93)	29 (91)	

* Time-to-TB treatment is defined as the time interval between date of inclusion and TB treatment start date (11 patients who never initiated TB treatment excluded).

Patients with clinically diagnosed TB (without bacteriological confirmation) and patients with positive results on lymph node aspirates (but without positive results in sputum) excluded.

The median culture TTP for smear-negative TB cases was 12 days (IQR, 8–14) and the median time-to-TB treatment was 20 days (IQR, 6–42). The median culture TTP was 8 days (IQR, 7–10) and the median time-to-treatment was 8 days (IQR, 3–18) for smear-positive TB cases. While 73 (90%) of culture-positive Xpert-positive TB patients started TB treatment within 2 months after inclusion, only 23 (55%) of culture-positive Xpert-negative TB patients started treatment during the same time interval (p<0.001) (Table 6).

Comparing Xpert-positive and Xpert-negative culture-positive TB patients, the significant difference in time-to-TB treatment did not translate into a significant difference in 2 month mortality (p = 0.70) (Table 6).

## Discussion

The intensified case finding approach applied in this investigation revealed a high prevalence (16.9%) of bacteriologically confirmed TB in HIV-positive adults residing in an uptake area in central Ethiopia. In only a minority of these cases had TB co-infection been considered prior to study inclusion. Importantly, these subjects were recruited in public health centers, which are the health facilities where increasing proportions of HIV-positive individuals in sub-Saharan Africa are managed. The rate of TB detected in our study is much greater than that found in the only previously published investigation on TB in HIV-positive adults performed in Ethiopia [29], and is in the range of prevalence figures reported from South Africa [14], [16], [37], considered to be among the highest for TB in HIV-positive individuals globally [38]. Although restricted to one geographical area, our findings suggest a considerable burden of unrecognized TB among HIV-positive individuals in Ethiopia, and highlight the need of improved diagnostic strategies that can also be used in facilities with limited resources.

Most evaluations of Xpert MTB/RIF have targeted patients with clinically suspected TB [20]. Yet, Xpert MTB/RIF is being considered for screening HIV-infected subjects for active TB in highly endemic settings [39], both due to greater TB prevalence among such persons, as well as due to increased TB disease severity. To our knowledge, the performance of Xpert MTB/RIF for TB screening in patients eligible to start ART has only been addressed previously in one published study from South Africa [14]. Overall, our findings were similar to those reported by Lawn et al [14], with an additional diagnostic yield using Xpert MTB/RIF in sputum of 47.4%, and an overall diagnostic performance of 66.4% (using liquid culture for reference). In agreement with other reports [14], [23], we also found correlations between Xpert MTB/RIF results and median culture TTP, as well as sputum smear status; reflecting a higher threshold of colony-forming units in the specimen for Xpert MTB/RIF detection as compared to liquid culture.

Interestingly, although paucibacillary manifestations of pulmonary TB are common in HIV-positive individuals [6], [40], patients with Xpert-negative culture-positive TB in our cohort had less advanced immunosuppression than those with a positive Xpert result. Smear-negative Xpert-positive patients had similar characteristics to those with smear-positive disease, and smear-positive TB was somewhat unexpectedly associated with more advanced immunosuppression. The overall sensitivity of Xpert MTB/RIF was also highest in the subset of patients with the most advanced immunosuppression (82.9% in patients with CD4 cell count ≤100 cells/mm^3^ as compared to 46.7% in patients with CD4 cell counts >200 cells/mm^3^). Although these findings are similar to those reported by Lawn et al [24], they are in contrast to other investigations on the association between bacterial burden and CD4 cell counts in HIV-infected subjects performed in TB clinics; in a South African study, the sensitivity for Xpert in subjects with CD4 cell counts below 200 cells/mm^3^ was lower compared to those with higher levels [23]. Similarly, higher bacillary density among smear-positive HIV-infected patients in Tanzania correlated with increasing CD4 cell levels [41]. One reason for this discrepancy could be that our screening approach led to detection of early-stage disease in subjects who had not yet developed symptomatic TB [5]. Asymptomatic (or subclinical) TB has been detected in considerable proportions of persons living in settings with high TB incidence, especially in HIV-positive individuals [37], [42].

Given the superior detection rate of Xpert MTB/RIF compared to smear microscopy, it has been suggested to introduce this method as a replacement for the latter technique [14]. Such a strategy has been estimated to be cost-effective in the South African context [23], but it is unclear whether this also applies for other countries on the continent [25].

Although the performance of sputum smear microscopy was clearly inferior to that of Xpert MTB/RIF in our cohort, this cheap, robust and well established method still detected 22.6% of TB patients. Furthermore, no false positive smear results were observed. In our opinion, Xpert MTB/RIF should rather be considered as an adjunct test for improved case detection in smear-negative HIV-positive individuals in low-income settings, instead of replacing smear microscopy. Our data indicate that testing a single morning sputum sample is sufficient for this purpose. In contrast to other studies on Xpert MTB/RIF performance [14], we did not find a significantly increased detection rate by using two instead of one sputum sample for Xpert MTB/RIF testing. This might be explained by the use of morning sputa (which have higher bacterial load in TB patients) instead of samples obtained in association with clinic visits (“spot samples”) [43].

Furthermore, clinical characterization of subsets of HIV-positive individuals who would benefit most from Xpert MTB/RIF testing could help to optimize testing algorithms in regions with restricted resources. However, the WHO clinical screening algorithm showed unsatisfactory specificity in this regard, since 78% of participants in our cohort without TB had a positive WHO symptom screen, illustrating the need for algorithms with greater specificity.

Our findings indicate that HIV/TB co-infected patients detected by Xpert MTB/RIF have more severe disease, both with regard to TB and HIV. Similar to a report from South Africa [24], cases missed by Xpert MTB/RIF did not show higher two-month mortality, despite significantly delayed initiation of TB treatment. The true impact on patient survival of Xpert screening is still difficult to estimate, since the outcome of persons with false negative Xpert results in a context without access to culture (and who would not receive TB treatment) might have been worse than observed here. Even if Xpert MTB/RIF testing provided rapid results, all Xpert-positive patients did not start TB treatment, mostly because of early loss to follow-up.

Twelve patients had positive Xpert MTB/RIF results but no bacterial growth in liquid culture. In line with revised WHO case definitions [36] we used sputum smear, culture or Xpert MTB/RIF as case definitions for TB. The significance of Xpert-positive culture-negative results has been discussed [23]; in accordance with other researchers, we do not consider such cases as false positive reactions [23], [44] One reason for this phenomenon could be that we used paired sputum samples for culture and Xpert MTB/RIF, and it is possible that the bacterial content in those samples (although obtained simultaneously) might have differed. This fact should also be taken into account when interpreting data on the performance of Xpert MTB/RIF performance using culture for reference.

Despite being executed by trained laboratory technologists, the occurrence of test failure was high (6.2% of samples tested). Apart from this observation, the requirement of stable electric power, temperature below 28°C and constant supply of consumables all represent major obstacles for the roll-out of Xpert MTB/RIF assay in the health systems of low-income countries.

This study was based on a large and well characterized cohort of HIV-infected adults, and provides new and much needed data on the TB prevalence among such individuals in Ethiopia. Our study participants were recruited from several public health centers providing ART in a defined uptake area, and we consider this to give more accurate estimates, both of the true TB prevalence in this context, as well as of the real-world performance of Xpert MTB/RIF. Determination of TB prevalence was based on several bacteriological diagnostic methods, using two paired morning sputum samples, as well as lymph node aspirates from patients with lymphadenopathy, which probably led to increased TB case detection in the study population.

In our study population, the majority of participants (both with and without final TB diagnosis) reported a positive WHO TB symptom screen. This illustrates the considerable overlap of disease symptoms in TB and HIV/AIDS. Although we did not specifically target subjects with suspected TB symptoms for this study, it is possible that the requirement of sputum samples for inclusion might have led to some bias with regard to the presence of airway symptoms. Since we did not collect other respiratory samples for mycobacterial diagnostic testing (eg. gastric aspirate or broncheo-alveolar lavage), we were unable to determine the rate of TB in the minor proportion of potential study participants who did not submit sputum samples (61/886; 6.9%), and whether this would have affected the overall prevalence figure. Furthermore, our study protocol did not include any diagnostic method for extrapulmonary manifestations of TB apart from peripheral lymphadenitis. This could have led to some underestimate of the prevalence of active TB in the population. The fact that we included patients who had initiated TB therapy during the preceding two weeks might have influenced culture positivity rates; still, the number of such cases was small, and only 3 of these 13 cases were culture-negative. This study did not aim to assess the prevalence and characteristics of drug resistance in detected TB strains, and phenotypic drug resistance patterns were not available. Data on MDR TB from Ethiopia are limited; reports suggest that the prevalence among patients without previous TB is low [27], and few of our participants reported prior TB treatment. Xpert MTB/RIF detected rifampicin resistance mutations in two cases (who were referred for further evaluation in tertiary centers in Addis Ababa). In settings with low prevalence of MDR TB it has been recommended to interpret positive Xpert results for rifampicin resistance with caution, since false positive reactions may occur [14], [45].

In conclusion, we found a high prevalence of previously undiagnosed TB in HIV-infected adults seeking care in health centers in central Ethiopia, illustrating the need of optimized screening strategies for TB in this population. Using Xpert MTB/RIF as part of such a screening algorithm in combination with smear microscopy would lead to increased case detection, especially in subjects with advanced immunosuppression. Yet, a significant subset of HIV-positive patients with active TB would still be missed using this technique, and the long-term outcomes of such individuals warrant further study. Furthermore, technical and cost-effectiveness issues have to be solved before general introduction of this diagnostic method can be considered.
